# Relevant SARS-CoV-2 Genome Variation through Six Months of Worldwide Monitoring

**DOI:** 10.1155/2021/5553173

**Published:** 2021-06-29

**Authors:** Abdelmalek Hakmaoui, Faisal Khan, Abdelhamid Liacini, Amanjot Kaur, Yacine Berka, Safaa Machraoui, Hafid Soualhine, Noureddine Berka, Hanane Rais, Brahim Admou

**Affiliations:** ^1^Center of Clinical Research, University Hospital Mohammed VI, Marrakech, Morocco; ^2^Cumming School of Medicine, University of Calgary, Canada; ^3^Immunogenetics Laboratory, Temple University and Hospital, Lewis Katz School of Medicine, USA; ^4^University of Calgary, Alberta, Canada; ^5^National Microbiology Laboratory Public Health Agency of Canada, 1015 Arlington Street, Winnipeg, MB, Canada; ^6^Department of Pathology and Lab Medicine, University of Calgary, Alberta, Canada; ^7^Bioscience Research Laboratory, Faculty of Medicine, Cadi Ayyad University, Marrakech, Morocco

## Abstract

Real-time genome monitoring of the SARS-CoV-2 pandemic outbreak is of utmost importance for designing diagnostic tools, guiding antiviral treatment and vaccination strategies. In this study, we present an accurate method for temporal and geographical comparison of mutational events based on GISAID database genome sequencing. Among 42523 SARS-CoV-2 genomes analyzed, we found 23202 variants compared to the reference genome. The Ti/Tv (transition/transversion) ratio was used to filter out possible false-positive errors. Transition mutations generally occurred more frequently than transversions. Our clustering analysis revealed remarkable hotspot mutation patterns for SARS-CoV-2. Mutations were clustered based on how their frequencies changed over time according to each geographical location. We observed some clusters showing a clear variation in mutation frequency and continuously evolving in the world. However, many mutations appeared in specific periods without a clear pattern over time. Various important nonsynonymous mutations were observed, mainly in Oceania and Asia. More than half of these mutations were observed only once. Four hotspot mutations were found in all geographical locations at least once: T265I (NSP2), P314L (NSP12), D614G (S), and Q57H (ORF3a). The current analysis of SARS-CoV-2 genomes provides valuable information on the geographical and temporal mutational evolution of SARS-CoV-2.

## 1. Introduction

The global pandemic of coronavirus disease (COVID-19) is caused by severe acute respiratory syndrome coronavirus-2 (SARS-CoV-2) [1]. The first genome sequencing of the virus was performed in humans on 07 January 2020 by Chinese scientists [2]. The genome of the virus contains 14 ORFs (open reading frames) encoding 27 proteins [3]. Approximately two-thirds of the viral RNA encode for 16 nonstructure proteins (NSPs) and are primarily located in the first ORF (ORF1a/b) of the viral genome. The remaining viral genome, located in the 3′-terminus of the SARS-CoV-2 genome, encodes for four essential structural proteins (spike (S) glycoprotein, small envelope (E), matrix (M), and nucleocapsid (N) proteins) [3, 4] and eight accessory proteins (3a, 3b, p6, 7a, 7b, 8b, 9b, and ORF) [3, 5]. It has been widely reported that the functional domains of the viral genome are linked to virulence, infectivity, ion channel formation, and virus release and that conserved functional domains across the species (likely 3a protein) are critical in the viral life cycle [6].

Current genome survey data suggests that single nucleotide variants (SNVs) are abundant, and a plentiful mutational diversity may define different major clusters of viruses now circulating globally [7]. Such observations raised the question of whether viral mutations are contributing to host tropism, changing antigenicity, and rapid global spread [8]. A recent study reported that SARS-CoV-2 has mutated in different patients, leading to the viral genome grouping into 6 genotypes [9]. In fact, the virus mutation rate drives viral evolution and genome variability [1].

The Ti/Tv ratio is a tool to measure the nucleotide mutation process and is an established component of substitution models [10]. The Ti/Tv ratio is computed as the ratio of the number of transition SNPs (purine-to-purine or pyrimidine-to-pyrimidine) to the number of transversion SNPs (purine-to-pyrimidine or vice versa). Estimation of the Ti/Tv ratio gives us a deeper understanding of the process of molecular evolution, which can help in modeling of the evolutionary process. The Ti/Tv ratio is also widely used [11, 12] as a quality control parameter for assessing the overall SNP quality.

Overall, close monitoring of the SARS-CoV-2 genome is important for identifying possible mutant strains and the potential impact of these mutations on the virulence and propagation of the virus between populations worldwide [13]. Genome efforts to track SARS-CoV-2 sequences have globally helped identify worrying variants.

Since December 2019, the GISAID database (https://www.gisaid.org) has provided a compilation of thousands of complete and partial SARS-CoV-2 genomes, contributed by clinicians and researchers from across the world. These sequencing data are useful in tracking intercontinental variation and the interpersonal evolutionary dynamics of the virus. Assessment of the virus sequence can provide scientific evidence to guide drug and vaccine development.

In the present investigation, we compared the mutational events of SARS-CoV-2 in six geographical areas (Africa, Asia, Europe, North America, Oceania, and South America) based on viral genome data available in the GISAID database, collected within a period of 6 months, from January to June 2020.

## 2. Materials and Methods

### 2.1. Source of Sequences and Exclusion Criteria

Genome sequences of SARS-CoV-2 were retrieved from the GISAID database (https://www.gisaid.org/) separately for six geographic locations (Africa, Asia, Europe, North America, Oceania, and South America) and for six periods of genome collection (01-31 January 2020, 01-29 February, 01-31 March, 01-30 April, 01-31 May, and 01-30 June). A full acknowledgment table of those labs which generated and uploaded data is provided as a supplementary file (available here). The sequences were filtered according to the following criteria: human host, complete genome (only genomes with >92000 pb), and high coverage (genomes containing less than 1% Ns and less than 0.05% of unique amino acid mutations without insertions or deletions). Only high-quality genomes with precise collection times were selected.

### 2.2. Mapping and SNP Calling

The SNP profile is a collection of SNP mutations with the corresponding nucleotide changes and positions in a genome. For each dataset, whole-genome sequences in FASTA format were mapped to reference genome MN908947.3, using the standard Geneious algorithm (Geneious Prime software 2020.2.1). To preserve sequences' site information, we trimmed end positions 1 to 55 and 29804 to 29903 as they are characterized by low coverage and a high rate of apparent sequencing or mapping errors. The “find variations/SNPs” tool in the Geneious software was used for SNP calling using reference genome NC045512.2 annotations obtained from the NCBI database. The data files in the CSV format were exported to an Excel file for further analysis.

### 2.3. Statistical Data Analysis

All statistical analyses and generation of plots were performed via RStudio software (version 1.3.959) using various package tools. Categorical variables were expressed as absolute frequencies and percentages. The nonparametric Wilcoxon test was used to compare the distribution of variant frequencies between the transition and transversion mutations, and the chi-squared test was used to compare the proportions of transition and transversion mutations between locations for each period and between periods for each location. A *p* value of less than 0.05 was considered statistically significant.

## 3. Results

### 3.1. Variant Filtration

A total of 42523 SARS-CoV-2 genomes derived from six geographical locations were fully analyzed from January 2020 to June 2020 (Table 1). The analysis showed a total of 23202 variants, which upon comparison with the reference genome showed 55% as transition mutations and 45% as transversion mutations. Upon analyzing all the variants, we observed that the Ti/Tv ratio was very low (Ti/Tv = 1.2) (Table 1). However, as we increased the cutoff value of variant frequencies, this ratio increased. At a cutoff of 1%, the Ti/Tv ratio became 3.2. At this level, only 55 variants were detected (79% were transition mutations, and 21% were transversion mutations). For the next step of our analysis, we considered only variants observed with a frequency over 1% in each dataset.

### 3.2. Characterization of SARS-CoV-2 Variants Geographically over Time

To understand the evolution of SARS-CoV-2 over the first six months of the pandemic, we analyzed data separately for each geographical location by categorizing the data according to the timing of sample collection as recorded in the GISAID database. As shown in Table 2, the largest proportion of genomes came from Europe (24326 samples, 57.2%), followed by North America (10878 samples, 25.6%), Asia (4305 sample, 10.1%), and Oceania (1904 samples, 4.5%), while genome sequences from Africa and South America accounted for only 2.6%, with 569 and 541 samples, respectively.

By focusing on hotspot mutations with frequencies over 1% in each dataset to filter out possible recurrent sequencing errors, we observed that transition mutations generally occurred more frequently than transversion mutations. Comparison of mutational characteristics of viral genomes from various geographical locations (Table 2) showed that the Ti/Tv ratios ranged from 2 to 5.5 and that the most common nucleotide change was the C>U transition (data not shown). The proportion of the latter ranged usually from 40% to 59%. The most common transversion was G>U whose proportion ranged from 9.23% to 27%. When comparing the distribution of Ti and Tv mutations for each geographical location at different time periods, we noticed no significant difference.

**Figure 1:**
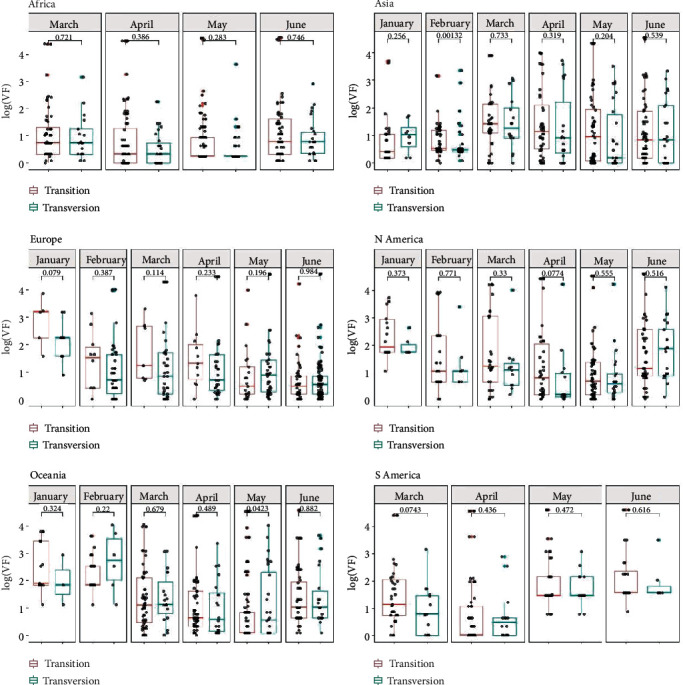
Boxplot comparing the distribution of transition and transversion mutations with variant frequencies according to the geographical location and period of sample collection using the nonparametric Wilcoxon test. The *x*-axis represents the mutation substitution type, and the *y*-axis represents the log-transformed variant frequency.

We then checked if the distribution of Ti and Tv mutations is associated with an increase in variant frequencies (Figure 1) and noted that there were more transversion mutations with low frequencies than with high frequencies. The Wilcoxon test showed a significant difference in the distribution of mutation frequencies between the Ti and Tv mutations in Asia in the February 2020 period with a *p* value of 0.0132. A significant difference was also observed in Oceania in May 2020 (*p* = 0.0423). For the rest of the datasets, there was no significant difference between the Ti and Tv mutations according to mutant frequencies.

### 3.3. Mutational Patterns of SARS-CoV-2

**Figure 2:**
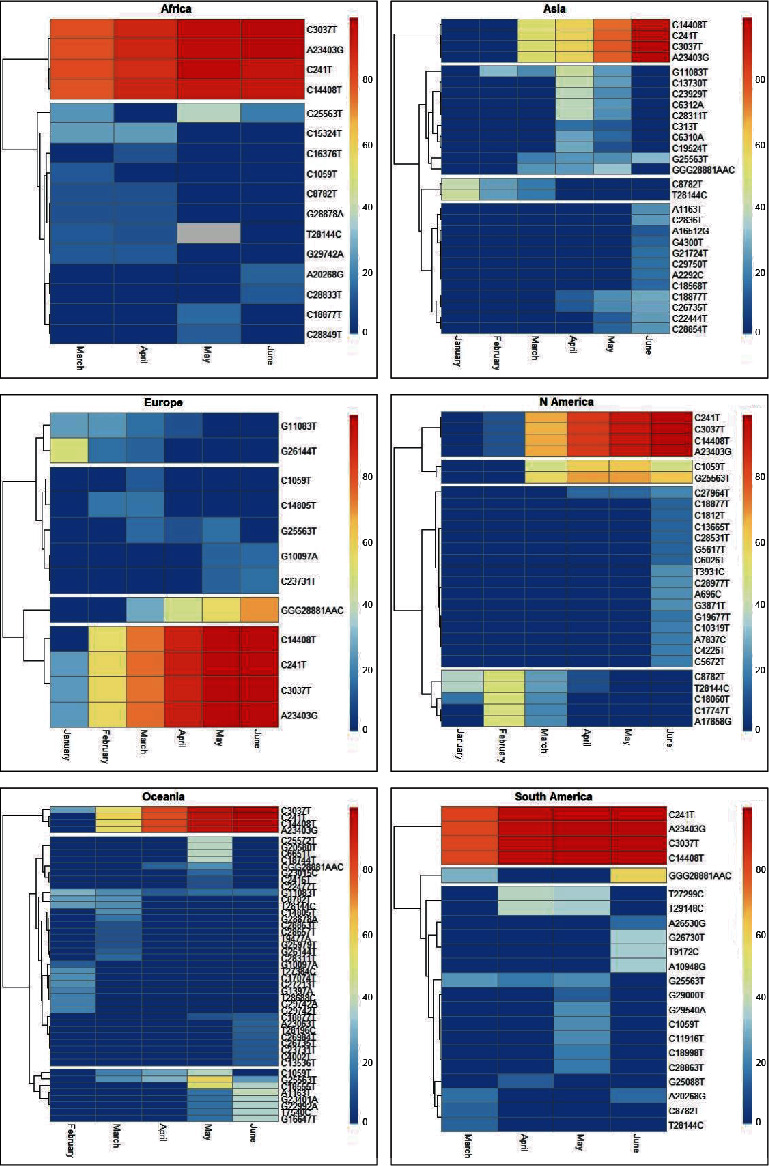
A heatmap showing the dynamics of hotspot mutation frequencies according to geographical locations. The period of collection is shown as rows, and hotspot mutations are shown as columns. Red/blue coloration implies higher/lower mutation frequencies.

To study the mutational patterns of SARS-CoV-2 across geographical locations, we selected the most abundant mutations defined as having a frequency of 10% or greater in at least one dataset. Heatmap analysis was performed to visualize the shared groups of mutations and to cluster them based on their frequency changes in chronological order (Figure 2). We found that four mutations (A23403G, C14408T, C241T, and C3037T) always cooccurred and showed the same clustering pattern in all geographical locations over time.

Our clustering analysis revealed that each geographical location was characterized by different mutational patterns and some of them were continuously evolving in the world. In Africa, only two groups can be clearly distinguished: the group of four mutations cited above and one group of 12 mutations without a clear temporal pattern. In Asia, the number of hotspot mutations was relatively low during January and February, but after March, more mutations were detected and more patterns were observed, with 4 groups of mutations. Interestingly, we noticed a cooccurrence of two mutations (C8782T and T28144C) that had appeared early in Asia and their frequencies declined thereafter. In Europe, two mutations clustered together (G11083T and G26144T) appeared with relatively higher frequency during the initial periods as compared to the later periods. We noted that the variant GGG mutated to AAC at position 28881–28883 and reached a frequency of more than 10% in March that increased over time. We identified another group of 5 mutations (C1059T, C14805T, G25563T, G10097A, and C2373T) without a clear pattern. Two of them were detected later during the May to June period (G10097A and C2373T). In North America, there were two identified groups of mutations, each divided into two subgroups. The first group was characterized by mutations with increasing frequencies over time, one subgroup of two mutations (G25563T and C1059T) and another subgroup of four mutations (A23403G, C14408T, C241T, and C3037T). The second group featured one subgroup with a large number of mutations (16 mutations) that appeared later, while the other subgroup featured mutations that emerged in the beginning, and then their frequencies declined during the May to July period. In Oceania, three mutational groups were identified. The first group was marked by the four mutations noticed in the other geographical locations. The second group was characterized by 35 mutations without a clear pattern, and the third group contained 8 mutations that emerged beginning in March (C1059T, G25563T, C18555T, A1163T, T7540C, G16647T, G22992A, and G2340A).

In South America, in addition to the clustering of A23403G, C14408T, C241T, and C3037T, we distinguished two groups of mutations, one of which featured 17 mutations gathered in the same cluster, while the other group contained the variant GGG mutated to AAC at position 28881–28883.

### 3.4. Nonsynonymous Substitutions and Associated Amino Acid Changes

**Figure 3:**
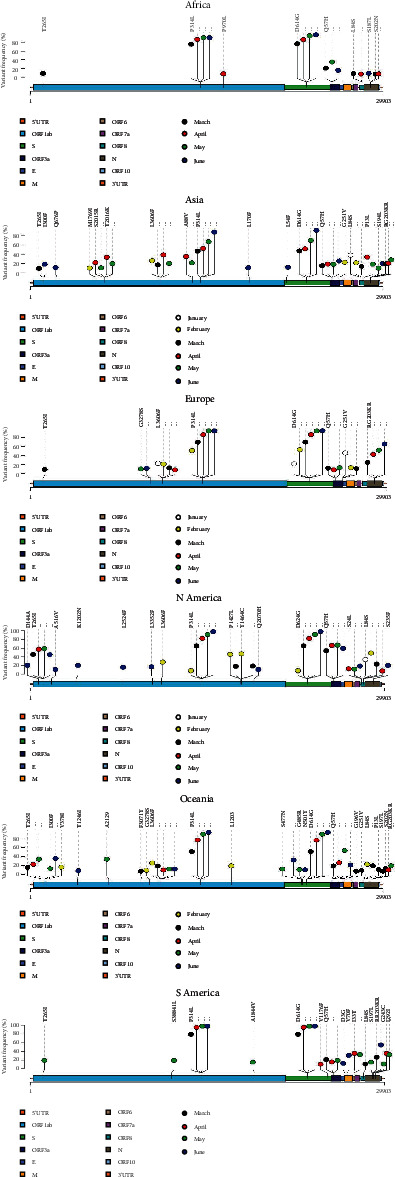
Lollipop plots showing the distribution of nonsynonymous hotspot mutations on the SARS-CoV-2 genome and the change of mutation frequencies over time within geographical locations. The presence of a mutation is shown on the *x*-axis (lollipop), and the frequency of mutations is shown on the *y*-axis vertical line. The period of genome collection is distinguished by color. Nucleotide coordinates are according to the SARS-CoV-2 reference genome. Amino acid positions are according to the mature peptides in the SARS-CoV-2 reference genome. Only nonsynonymous mutations with frequencies ≥ 10% are presented here.

Our analysis revealed a difference between geographical locations in the number of sites possessing hotspot nonsynonymous mutations with frequencies ≥ 10% at least once (Figure 3). The distribution of these mutations varied widely among different proteins of SARS-CoV-2. The highest number of mutations was observed in Oceania (*n* = 22), followed by Asia (*n* = 18), North America (*n* = 16), and South America (*n* = 15). The lowest number was observed in Europe and Africa with 8 mutations observed for both regions. About 47% of these mutations were observed only once. Four hotspot substitutions were found in all locations at least once: T265I (NSP2), P314L (NSP12), D614G (S), and Q57H (ORF3a). In Oceania, we have also observed three substitutions (S477N, G485R, and N501Y) in addition to the D614G substitution on the S protein.

## 4. Discussion

The use of advanced sequencing technologies has the potential of producing multiple-time point whole-genome data, which provides insight into the evolution of the SARS-CoV-2 genome during the COVID-19 pandemic in each geographical area, and in identifying a comprehensive list of candidate adaptive mutations for this stage of the pandemic. It is important to note that the viral genome data available are geographically biased in favor of regions performing extensive sequencing. The highest number of samples was observed in Europe, followed by North America, Asia, and then Oceania, while the lowest data were observed in Africa and South America.

Comparison of whole-genome samples with the reference genome revealed a number of mutations occurring mostly at low frequencies. Mutations with very low frequencies are likely due to errors in the NGS procedure rather than true variants in viral strains [14, 15]. This can be explained by the fact that Ti/Tv ratios increase when variants with low frequencies are removed. During six months of monitoring, we found 55 variants that have been detected with a frequency greater than 1%. However, Callaway [16] reported that a typical SARS-CoV-2 virus accumulates only two single-letter mutations per month in its “genome.”

In practice, Ti/Tv ratios can be used to determine which threshold should be used in QC data [11]. Rayko and Komissarov [17] have reported a lower Ti/Tv ratio in the unique genome variation of SARS-CoV-2. In the case of a random distribution of Ti and Tv mutations (i.e., without any biological influence), a ratio of 0.5 would be expected, simply due to the fact that there are twice as many Tv mutations possible as Ti. However, in the biological context, a bias of Ti versus Tv is generally observed as a function of unequal base frequencies. In fact, this Ti-Tv substitution bias has been noted in both the eukaryotic and prokaryotic genomes [18, 19]. Using a cutoff filtering frequency of 1% and separately analyzing our data by geographical location and time period, we generally observed a Ti versus Tv bias, which was previously reported by some authors [20].

Generally, the predominant substitution was C>U transition, which might be intervened by cytosine deaminases [21]. Multiple groups have observed the predominance of C to T (U) substitutions in SARS-CoV-2 [22, 23]. The high frequency of C>U transitions likely reflects the virus adaptation processes in its hosts [20]. Surprisingly, G>U transversions were also frequent. Panchin and Panchin [24] reported a 9-fold excess of G>U transversions among SARS-CoV-2 mutations over relative substitution frequencies between SARS-CoV-2 and a close relative coronavirus from bats (RaTG13), suggesting that the mutational patterns of SARS-CoV-2 could have changed after transmission to humans.

When comparing the distribution of the proportions of transition and transversion mutations between different geographical locations and time periods, we noted that the bias in transition versus transversion mutation in SARS-CoV-2 is not associated with geographical and temporal effects.

Heatmap analysis (Figure 2) revealed remarkable hotspot mutation patterns for SARS-CoV-2. Mutations were clustered based on how their frequencies changed over time according to each geographic location. We noticed that some clusters show a clear increase or decrease in mutation frequency. However, many mutations appeared in specific periods without a clear pattern over time. Other studies reported that only a handful of clusters were prevalent in the early stage of the pandemic, and even some mutations arose independently. As shown in our study, continuous mutations accumulate in transmitted strains, and the emergence of small clusters was replaced quickly, whereas others gradually became dominant because the mutations were fixed, such as the D614G mutation in the RBD region. It is worth noting that the D614G mutation at position 23403 always accompanies the three most frequent mutation sites in the ORF1ab (14408 in NSP12 and 3037 in NSP3) region, as well as the mutation at position 241. The four mutations occurred probably during the transition between the first cluster reported in Wuhan and the subsequent clusters that spread globally. Moreover, researchers reported that the increase of these mutations was the result of a fitness advantage rather than a genetic drift [25] of the virus by increasing its infectivity [26]. We assume that the cooccurrence of this group of mutations can functionally cooperate in the stability, transmission, and adaptability of the virus [27].

Another group of mutations showing a constant increase in frequency was the three adjacent nonsynonymous mutations in the N protein, and the nucleotide sequence GGG changed to AAC, resulting in a change of amino acid from RG to KR (AGGGGA coding for RG changed to AAACGA coding for KR). This group of mutations was identified first in February in Europe and then in other locations.

Two mutations clustered together in North America (G25563T and C1059T) appeared in March with a medium frequency and remained so until June. Both mutations also appeared in all geographical locations in at least one period but with lower frequency than in North America. The C1059T mutation causing amino acid substitutions (T265I) is in gene regions of NSP2 (ORF1ab), whereas the G25563T mutation is on the gene region of ORF3a, which encodes for the largest protein in the SARS-related CoV accessory family proteins. The product of ORF3a is a unique membrane protein and is essential for the pathogenesis of the disease [2, 6].

An interesting mutational pattern was observed mainly in geographical locations performing extensive sequencing. It concerns mutations that appeared early and whose frequencies subsequently declined. In Asia, two mutations cooccurred, the C8782T silent mutation in the NSP4 gene and the T28144C nonsynonymous mutation (L84S) in the ORF8 gene. There is a controversial debate about the ancestry of L and S types of this mutation and its functional impact [28, 29]. During the initial stages of the outbreak in Wuhan, a decrease in the frequency of L-type mutations was observed after January 2020, suggesting that 84S may exhibit some advantages over 84 L. Studies suggest that 84S may lead to structural disorders in the C-terminus of the protein and may also produce a new phosphorylation target for serine/threonine kinases of mammalian hosts.

The same pattern was observed in Europe for two transversion mutations: the G11083T, leading to an L3606F change within the NSP6 protein in ORF1a, and the G26144T, leading to a G251V change in ORF3a. The frequency of the G251V substitution was estimated at 48% in Europe in January and decreased to 13% in March (Figure 3), while the emergence of this substitution is consistent with the lockdown of Wuhan on 23 January 2020 [30].

Along with the two mutations observed in Asia (C8782T, T28144C), a cluster of three other mutations (C18060T, C17747T, and A17858G) stood out in North America. The complete disappearance of this cluster was observed during the May to June period. The C18060T change is a silent mutation in the ORF1b gene. However, the two other mutations are nonsynonymous, leading to P1427L and Y1464C changes in NSP13 (helicase) which catalyzes the unwinding of duplex oligonucleotides into single strands. According to Guan et al. [29], the substitution of proline with leucine (P1427L) is not expected to create noticeable effects. Y1464 is part of a region that contributes to the binding and unwinding of duplex oligonucleotides [31]. Its substitution by cysteine would decrease the stability and enhance the dynamics of this particular region and possibly affect the binding and processing of RNA [29].

Various nonsynonymous mutations were observed during the monitoring period. These can be due to genetic evolution and adaptation including the infectivity and pathogenicity of SARS-CoV-2. However, minimal mutations in the genome, represented by a single D614G mutation in the S protein, have the capacity to alter the traits of a protein, affecting the virus infectivity and clinical outcomes, as well as the epidemiology of the virus [16, 32]. The D614G mutation in the spike (S) protein and P314L in the nonstructural protein 12 (NSP12) are consistently related and common in all geographic locations with increasing frequency over time. The spike region determines the specific binding of SARS-CoV-2 to the host receptor and the initiation of viral replication; this region is reported to be the most potent and essential for viral attachment and entry into the host cells [33]. Eaaswarkhanth et al. [34] speculate that the S D614G strains could be more virulent, increasing the severity of the virus in infected individuals, especially in Europe where this mutation is prominent [35]. The P314L mutation found within the RNA-dependent polymerase may play a causal role in enhanced viral replication and therefore should be considered for potential contribution to infectivity [36].

The SARS-CoV-2 virus is continuously evolving and has already formed heterogenic clusters. In addition, several fixed mutations have also been observed in different continents. These mutations can be essential for the adaptation of the virus to the human host. T265I and Q57H substitutions are likely to be associated with pathogenesis. Indeed, NSP2 involves mitochondrial biogenesis and intracellular signaling while ORF3a can induce cell apoptosis [37, 38]. In the spike protein region, three other important nonsynonymous mutations other than D614G have been observed in Oceania (S477N, G485R, and N501Y). This requires further investigation, in particular for possible vaccine application. Amino acid changes may play an important role in increasing the virulence of viral strains by inducing conformational changes in discontinuous neutralizing epitopes [35].

Some mutations could be the result of the virus adapting to specific environmental conditions in a given geographic area, such as the climate [39]. However, the potential geoclimatic effects on the mutations observed must be evaluated by clinical and/or experimental studies.

### 4.1. Study Limitations

This study has some limitations, such as the lack of clinical patient data, as well as unbalanced sample sizes between different geographic areas.

In addition, the sequenced viral genomes are not randomly selected from the global population and are therefore susceptible to bias.

## 5. Conclusion

In summary, our data provide valuable information on the geographic and temporal genome evolution of SARS-CoV-2. The mutations observed vary according to the geographical distribution and the period of monitoring of the virus. This variation would be influenced by the virus-host interaction. Beside these mutational events resulting in the transformation to a more virulent strain, there are a number of highly conserved regions in the SARS-CoV-2 genome which could be utilized as potential targets for inhibitory drugs and in vaccine development. Effective and timely genome surveillance of viral sequences is worthwhile for effective prevention and control.

## Figures and Tables

**Table 1:** Distribution of the number of transition and transversion mutations and the Ti/Tv ratio according to cutoffs of variant frequencies (VF) using global data of 42523 SARS-CoV-2 genomes.

Cutoff frequency	Transition	Transversion	Ti/Tv ratio
VF ≥ 1%	42 (79%)	13 (21%)	3.2
VF ≥ 0.1%	832 (73%)	303 (27%)	2.7
VF ≥ 0.01%	5423 (67%)	3225 (37%)	1.7
Total	12822 (55%)	10380 (45%)	1.2

**Table 2:** Distribution of the number of genome samples (*N*) and the number of transition (Ti) and transversion (Tv) mutations and the Ti/Tv ratio for data from each location during different periods of virus collection. A chi-squared test was used to compare the distribution of mutation types by period for each location and to compare the mutation type by location for each period. NA: data not available or sample number under 20.

Period of collection	January	February	March	April	May	June	*p* value
Africa (*N* = 569)							0.9134
*N*	NA	NA	142	210	78	139	
Ti, *n* (%)	NA	NA	64 (76.19)	80 (72.07)	139 (73.54)	67 (72.04)	
Tv, *n* (%)	NA	NA	20 (23.81)	31 (27.93)	50 (26.46)	26 (27.96)	
Ti/Tv			3.2	2.58	2.78	2.58	

Asia (*N* = 4305)							0.8918
*N*	379	551	1247	1025	685	418	
Ti, *n* (%)	30 (78.95)	36 (72)	38 (70.37)	44 (69.94)	56 (68.3)	65 (73.03)	
Tv, *n* (%)	8 (21.05)	14 (28)	16 (29.63)	19 (30.16)	26 (31.7)	24 (26.97)	
Ti/Tv	3.75	2.57	2.37	2.32	2.15	2.7	

Europe (*N* = 24326)							0.1793
*N*	21	196	10025	10450	3003	631	
Ti, *n* (%)	86 (71.67)	55 (84.62)	37 (80.43)	33 (75)	42 (66.67)	117 (70.9)	
Tv, *n* (%)	34 (28.33)	10 (15.38)	9 (19.57)	11 (25)	21 (33.33)	48 (29.02)	
Ti/Tv	2.53	5.5	4.11	3	2	2.34	

North America (*N* = 10878)							0.7178
*N*	20	106	5370	3858	1051	473	
Ti, *n* (%)	18 (75)	91 (77.75)	32 (74.42)	42 (75)	72 (76.6)	61 (74.39)	
Tv, *n* (%)	6 (25)	26 (22.22)	11 (25.58)	14 (25)	22 (23.4)	21 (25.61)	
Ti/Tv	3	3.5	2.90	3	3.27	2.90	

Oceania (*N* = 1904)							0.2222
*N*	NA	32	1315	362	88	107	
Ti, *n* (%)	NA	26 (82.53)	57 (75)	61 (75.31)	132 (72.13)	185 (68.01)	
Tv, *n* (%)	NA	6 (17.65)	19 (25)	20 (24.61)	51 (27.87)	87 (31.99)	
Ti/Tv		4.67	3	3.05	2.59	2.12	

South America (*N* = 541)							0.6071
*N*	NA	NA	289	207	23	22	
Ti, *n* (%)	NA	NA	39 (72.22)	58 (80.56)	53 (75.71)	32 (82.1)	
Tv, *n* (%)	NA	NA	15 (27.78)	14 (19.44)	17 (24.29)	7 (17.9)	
Ti/Tv	NA	NA	2.6	4.14	3.12	4.6	
*p* value	0.6654	0.4094	0.8990	0.77753	0.68839	0.52399	

## Data Availability

Related supplementary data of the current manuscript are available upon request.

## Supplementary Materials

Supplementary Materials Acknowledgment table.

## References

[1] Pachetti M., Marini B., Benedetti F. (2020). Emerging SARS-CoV-2 mutation hot spots include a novel RNA-dependent-RNA polymerase variant. *Journal of Translational Medicine*.

[2] Lu R., Li J., Niu P. (2020). Genomic characterisation and epidemiology of 2019 novel coronavirus: implications for virus origins and receptor binding. *The Lancet*.

[3] Wu A., Peng Y., Huang B. (2020). Genome composition and divergence of the novel coronavirus (2019-nCoV) originating in China. *Cell Host & Microbe*.

[4] Guo Y.-R., Cao Q. D., Hong Z. S. (2020). The origin, transmission and clinical therapies on coronavirus disease 2019 (COVID-19) outbreak - an update on the status. *Military Medical Research*.

[5] Wang H., Li X., Zhang S. (2020). The genetic sequence, origin, and diagnosis of SARS-CoV-2. *European Journal of Clinical Microbiology & Infectious Diseases*.

[6] Issa E., Merhi G., Panossian B., Salloum T., Tokajian S. (2020). SARS-CoV-2 and ORF3a: nonsynonymous mutations, functional domains, and viral pathogenesis. *mSystems*.

[7] Yao H., Lu X., Chen Q. (2020). Patient-derived mutations impact pathogenicity of SARS-CoV-2. *medRxiv*.

[8] Phan T. (2020). Genetic diversity and evolution of SARS-CoV-2. *Infection, Genetics and Evolution*.

[9] Zhang L., Shen F.-M., Chen F., Lin Z. (2020). Origin and evolution of the 2019 novel coronavirus. *Clinical Infectious Diseases*.

[10] Kimura M. (1980). A simple method for estimating evolutionary rates of base substitutions through comparative studies of nucleotide sequences. *Journal of Molecular Evolution*.

[11] Guo Y., Zhao S., Sheng Q. (2014). Multi-perspective quality control of Illumina exome sequencing data using QC3. *Genomics*.

[12] 1000 Genomes Project Consortium, Abecasis G. R., Altshuler D. (2010). A map of human genome variation from population-scale sequencing. *Nature*.

[13] Abdullahi I. N., Emeribe A. U., Mustapha J. O. (2020). Exploring the genetics, ecology of SARS-COV-2 and climatic factors as possible control strategies against COVID-19. *Le Infezioni in Medicina*.

[14] Beerenwinkel N., Günthard H. F., Roth V., Metzner K. J. (2012). Challenges and opportunities in estimating viral genetic diversity from next-generation sequencing data. *Frontiers in Microbiology*.

[15] Bamford C., Wignall-Fleming E., Sreenu V. B., Randall R., Duprex P., Rima B. (2019). Unusual, stable replicating viruses generated from mumps virus cDNA clones. *PloS One*.

[16] Callaway E. (2020). The coronavirus is mutating - does it matter?. *Nature*.

[17] Rayko M., Komissarov A. (2020). Quality control of low-frequency variants in SARS-CoV-2 genomes. *bioRxiv*.

[18] Duchêne S., Ho S. Y., Holmes E. C. (2015). Declining transition/transversion ratios through time reveal limitations to the accuracy of nucleotide substitution models. *BMC Evolutionary Biology*.

[19] Rosenberg M. S., Subramanian S., Kumar S. (2003). Patterns of transitional mutation biases within and among mammalian genomes. *Molecular Biology and Evolution*.

[20] Matyášek R., Kovařík A. (2020). Mutation patterns of human SARS-CoV-2 and bat RaTG13 coronavirus genomes are strongly biased towards C>U transitions, indicating rapid evolution in their hosts. *Genes*.

[21] Frederico L. A., Kunkel T. A., Shaw B. R. (1993). Cytosine deamination in mismatched base pairs. *Biochemistry*.

[22] Koyama T., Platt D., Parida L. (2020). Variant analysis of COVID-19 genomes.

[23] Wang C., Liu Z., Chen Z. (2020). The establishment of reference sequence for SARS-CoV-2 and variation analysis. *Journal of Medical Virology*.

[24] Panchin A. Y., Panchin Y. V. (2020). Excessive G-U transversions in novel allele variants in SARS-CoV-2 genomes. *PeerJ*.

[25] Plante J. A., Liu Y., Liu J. (2021). Spike mutation D614G alters SARS-CoV-2 fitness. *Nature*.

[26] Bai Y., Jiang D., Lon J. R. (2020). Comprehensive evolution and molecular characteristics of a large number of SARS-CoV-2 genomes reveal its epidemic trends. *International Journal of Infectious Diseases*.

[27] Laha S., Chakraborty J., Das S., Manna S. K., Biswas S., Chatterjee R. (2020). Characterizations of SARS-CoV-2 mutational profile, spike protein stability and viral transmission. *Infection, Genetics and Evolution*.

[28] Chaw S.-M., Tai J.-H., Chen S.-L. (2020). The origin and underlying driving forces of the SARS-CoV-2 outbreak. *Journal of Biomedical Science*.

[29] Guan Q., Sadykov M., Mfarrej S. (2020). A genetic barcode of SARS-CoV-2 for monitoring global distribution of different clades during the COVID-19 pandemic. *International Journal of Infectious Diseases*.

[30] Ceraolo C., Giorgi F. M. (2020). Genomic variance of the 2019-nCoV coronavirus. *Journal of Medical Virology*.

[31] Jia Z., Yan L., Ren Z. (2019). Delicate structural coordination of the severe acute respiratory syndrome coronavirus Nsp13 upon ATP hydrolysis. *Nucleic Acids Research*.

[32] Li Q., Wu J., Nie J. (2020). The impact of mutations in SARS-CoV-2 spike on viral infectivity and antigenicity. *Cell*.

[33] Huang Y., Yang C., Xu X.-F., Xu W., Liu S.-W. (2020). Structural and functional properties of SARS-CoV-2 spike protein: potential antivirus drug development for COVID-19. *Acta Pharmacologica Sinica*.

[34] Eaaswarkhanth M., Al Madhoun A., Al-Mulla F. (2020). Could the D614G substitution in the SARS-CoV-2 spike (S) protein be associated with higher COVID-19 mortality?. *International Journal of Infectious Diseases*.

[35] Cao Y. C., Yeung W. S., Law M., Bi Y. Z., Leung F. C., Lim B. L. (1998). Molecular characterization of seven Chinese isolates of infectious bursal disease virus: classical, very virulent, and variant strains. *Avian Diseases*.

[36] Korber B., Fischer W. M., Gnanakaran S. (2020). Tracking changes in SARS-CoV-2 spike: evidence that D614G increases infectivity of the COVID-19 virus. *Cell*.

[37] Zhang Z., Shen L., Gu X. (2016). Evolutionary dynamics of MERS-CoV: potential recombination, positive selection and transmission. *Scientific Reports*.

[38] Cornillez-Ty C. T., Liao L., Yates J. R., Kuhn P., Buchmeier M. J. (2009). Severe acute respiratory syndrome coronavirus nonstructural protein 2 interacts with a host protein complex involved in mitochondrial biogenesis and intracellular signaling. *Journal of Virology*.

[39] Pietro R. D., Basile M., Antolini L., Alberti S. (2020). Genetic drift and environmental spreading dynamics of COVID-19. *medRxiv*.

